# HDAC5-mediated deacetylation and nuclear localisation of SOX9 is critical for tamoxifen resistance in breast cancer

**DOI:** 10.1038/s41416-019-0625-0

**Published:** 2019-11-06

**Authors:** Yue Xue, Wenwen Lian, Jiaqi Zhi, Wenjuan Yang, Qianjin Li, Xingyi Guo, Jiahao Gao, Hao Qu, Weiqiang Lin, Zhongqi Li, Lihua Lai, Qingqing Wang

**Affiliations:** 1 Institute of Immunology, Zhejiang University School of Medicine, Hangzhou, China; 2 Division of Epidemiology, Department of Medicine, Vanderbilt Epidemiology Center, and Vanderbilt-Ingram Cancer Center, Vanderbilt University School of Medicine, Nashville, TN USA; 3 Department of Orthopedic Surgery, The Second Affiliated Hospital of Zhejiang University School of Medicine, Hangzhou, China; 4 The Kidney Disease Center, The First Affiliated Hospital, Institute of Translational Medicine, Zhejiang University School of Medicine, Hangzhou, China; 5 Department of Surgical Oncology, The First Affiliated Hospital, Zhejiang University School of Medicine, Hangzhou, China; 6 Department of Pharmacology, Zhejiang University School of Medicine, Hangzhou, China

**Keywords:** Breast cancer, Acetylation

## Abstract

**Background:**

Tamoxifen resistance remains a significant clinical challenge for the therapy of ER-positive breast cancer. It has been reported that the upregulation of transcription factor SOX9 in ER^+^ recurrent cancer is sufficient for tamoxifen resistance. However, the mechanisms underlying the regulation of SOX9 remain largely unknown.

**Methods:**

The acetylation level of SOX9 was detected by immunoprecipitation and western blotting. The expressions of HDACs and SIRTs were evaluated by qRT-PCR. Cell growth was measured by performing MTT assay. ALDH-positive breast cancer stem cells were evaluated by flow cytometry. Interaction between HDAC5 and SOX9 was determined by immunoprecipitation assay.

**Results:**

Deacetylation is required for SOX9 nuclear translocation in tamoxifen-resistant breast cancer cells. Furthermore, HDAC5 is the key deacetylase responsible for SOX9 deacetylation and subsequent nuclear translocation. In addition, the transcription factor C-MYC directly promotes the expression of HDAC5 in tamoxifen resistant breast cancer cells. For clinical relevance, high SOX9 and HDAC5 expression are associated with lower survival rates in breast cancer patients treated with tamoxifen.

**Conclusions:**

This study reveals that HDAC5 regulated by C-MYC is essential for SOX9 deacetylation and nuclear localisation, which is critical for tamoxifen resistance. These results indicate a potential therapy strategy for ER^+^ breast cancer by targeting C-MYC/HDAC5/SOX9 axis.

## Background

Although endocrine therapy by using oestrogen antagonist tamoxifen can effectively improve the survival rates of oestrogen receptor-alpha (ERα) positive breast cancer patients, however a large proportion of patients are resistant to tamoxifen therapy. Tamoxifen resistance remains a significant clinical obstacle, and there is an urgent need to clarify the relapse mechanisms after the endocrine therapy.^1^

Recently, cancer stem cell-related transcription factor SOX9 located in the nucleus has been shown to cause endocrine-therapy resistance.^2^ SOX9 belongs to SRY-related high-mobility-group box (SOX) protein family, which is comprised by a group of transcriptional regulators that have a highly conserved high-mobility group (HMG) domain similar to that of sex-determining region Y protein (SRY) that mediates DNA binding function.^3,4^ Several SOX proteins have been identified as critical transcription factors (TFs) involved in embryonic development and cell fate decision,^5–8^ such as SOX9, which was reported to be essential for cartilage differentiation. Cartilage cells with cytoplasmic SOX9 exhibit impaired transcriptional activation of two well-characterised SOX9 target genes, including collagen type IIa1 and β-catenin for chondrocyte differentiation.^9^ In addition, SOX9 is also involved in tumour invasion and metastasis, attenuating therapeutic sensitivity and promoting stem cell proliferation. SOX9 enhances the liver cancer stem cells (CSCs) self-renew through maintaining non-symmetry cell dividing.^10^ In human breast cancer, previous study has shown that cytoplasmic accumulation of SOX9 is significantly correlated with enhanced proliferation in invasive ductal carcinoma and metastatic breast cancer.^11,12^ However, in ER^+^ breast cancer cell, SOX9 is localised in the nucleus.^2^ Notably, SOX9 nuclear expression is upregulated in tamoxifen resistant breast cancer (TAMR) cell that is sufficient to cause endocrine resistance.^2^ Studies have shown that SIRT1-mediated deacetylation of SOX9 renders its nuclear localisation, which sequentially facilitates SOX9 binding to the promoters of its target genes.^13–15^ However, so far, how SOX9 subcellular localisation is regulated remains largely unknown, especially in TAMR cells.

Protein acetylation and deacetylation have been defined as important post-translational modification processes for the activity, stability and subcellular localisation of proteins. HDACs constitute a family of protein deacetylases that remove acetyl groups from lysine residues. Human HDACs are grouped into five classes based on their similarity to known yeast factors. Class I and class III HDACs are respectively similar to the yeast transcriptional repressor yRPD3 and ySIR2, while class IIa and IIb HDACs are similar to yHDA1.^16^ Class IV contains just one isoform (HDAC11), which is not highly homologous with any of the yeast enzymes.^17^ HDAC5, belonging to class IIa HDACs and can shuttle between the nucleus and cytoplasm, has been implicated in many biological processes.^16^ As a repressor of angiogenesis, HDAC5 regulates the expression of angiogenesis-related genes in endothelial cells.^18^ In addition, HDAC5 is also found to be involved in regulating basal type of breast cancer cells proliferation and therapeutic resistance.^19^ Mechanistically, HDAC5 increases survivin and miR-125a-5p expression, leading to tamoxifen resistance in ER^+^ breast cancer cells.^20^

In this study, we demonstrated the indispensable role of HDAC5 in mediating the deacetylation and nuclear localisation of SOX9 in tamoxifen resistant breast cancer cells. Furthermore, the C-MYC activation in TAMR cells markedly increases the expression of HDAC5. Therefore, our findings identify that the C-MYC–HDAC5–SOX9 axis is an eminently potential target for intervention of tamoxifen resistant breast cancer cells.

## Methods

### Reagents and antibodies

Rabbit polyclonal anti-SOX9 (Millipore, AB5535), Mouse anti-Flag tag (Abmart, M20008), Mouse anti-MYC tag (Abmart, M20012), Mouse monoclonal anti-GAPDH (Beyotime, AF0006), Rabbit polyclonal anti-HDAC5 (CST, 20458), Rabbit anti-C-MYC (D3N8F) (CST, 9402), Rabbit monoclonal Anti-C-MYC (phospho S62) (Abcam, ab185655), Rabbit anti-Di-Methyl-Histone H3 (Lys27) (CST, 9728), Anti-Acetylated Proteins antibody (Abcam, ab193), Rabbit anti-Akt (pan) (C67E7) (CST, 4691), Rabbit anti-phospho-AKT (CST, 13038), Rabbit anti-p38 MAPK (D13E1) (CST, 9212), Rabbit anti-phospho-p38 MAPK (Thr180/Tyr182) (3D7) (CST, 9215), Rabbit anti-phospho-p44/42 MAPK (Erk1/2) (Thr202/Tyr204) (CST, 4370), Rabbit anti-p44/42 MAPK (Erk1/2) CST 4695 Rabbit anti-Akt (pan) (C67E7) (CST, 4691), Rabbit anti-Src (36D10) (CST, 2109), Rabbit anti-phospho-Src (Tyr416) (CST, 6943), Mouse anti-HER2 (Invitrogen, AHO1011), phospho-HER2 (Tyr1248) (CST, #2247), Rabbit anti-ABCG2 (CST, 42078), Rabbit anti-ERα (CST, 8644), Rabbit anti-Bcl2 (Proteintech, 12789-1-AP), Rabbit anti-p21 (Proteintech, 10355-1-AP), Rabbit anti-ALDH1A1 (CST, 36671T), Rabbit anti-Sox2 (CST, 3579S), Mouse anti-CD44 (CST, 3570), Rabbit anti-Flag-M2 Magnetic Beads (Sigma, M8823), Goat anti-Rabbit IgG (H + L) Secondary Antibody, Alexa Fluor 594 (Invitrogen, R37117), Goat anti-Rabbit IgG (H + L) Highly Cross-Adsorbed Secondary Antibody, Alexa Fluor 488 (Invitrogen, A-11034), Thiazolyl Blue Tetrazolium Bromide (Sigma M2128), ALDEFLUOR™ Kit (STEM CELL, #01700), Dual luciferase assay (Promega, 0000170721) and SimpleChIP Assay Kits (CST, 9005): MS275, EX527 and LMK-235 (Selleck).^21^

### Cell lines and cell culture

MCF-7, T47D and MDA-MB-231 cells were cultured by standard procedures of American Type Culture Collection. MCF-7, MDA-MB-231 and T47D cells were maintained in DMEM medium, supplemented with 10% foetal bovine serum under humidified atmosphere containing 5% CO_2_ at 37 °C. For TAMR T47D cells, T47D cells were cultured with tamoxifen for 20 passages with tamoxifen (6 μm).^22^ For TAMR MCF-7 cells, MCF-7 was cultured with tamoxifen (1 μm) for 12 months.^23^

### Quantification of ALDH-positive cells

The ALDEFLUOR kit was used to determine the percentage of cells with high ALDH activity following the manufacturer’s protocol (ALDEFLUOR Stem Cell Technologies, 01700). In brief, cells were washed with phosphate-buffered saline and re-suspended in ALDEFLUOR assay buffer (2 × 10^5^ cells/ml) and incubated with ALDEFLUOR substrate with or without ALDH inhibitor, diethylaminobenzaldehyde (DEAB) for 45 min at 37 °C in a water bath. Fluorescence-activated cells were analysed and determined the percentage of ALDH-positive cells in the samples.

### Immunofluorescence

The cells were washed three times with PBS and fixed for 15 min at room temperature with 4% (vol/vol) paraformaldehyde. The primary antibody, anti-SOX9 (1:250 dilution, Millipore), was used. Fixed cells were rinsed with PBS and then were incubated for 10 min on ice with 0.2% Triton X-100 and 0.2% BSA in PBS. Following punching, nonspecific binding in the cells was blocked by incubation for 30 min at room temperature with 0.3% Triton X-100 and 5% BSA in PBS, and cells were incubated overnight at 4 °C with specific primary antibodies (identified above). After three washes with PBS, the cells were incubated for another 2 h with secondary antibodies. Subsequently, the cells were washed three times with PBS and were stained with PBS containing DAPI (Sigma). All images were collected with a confocal microscope (Nikon A1R, Japan).

### Immunoblot and immunoprecipitation assays

For immunoprecipitation, whole-cell extracts were lysed in IP Lysis Buffer (Pierce, 87785) and a protease inhibitor ‘cocktail’ (Sigma, P8340). Cell lysates were centrifuged for 15 min at 12,000 × *g*. Supernatants were collected and incubated with protein G magnetic beads (Invitrogen, 00455858) together with specific antibodies. After overnight incubation, protein G magnetic beads were washed five times with IP wash buffer. Immunoprecipitation was eluted by SDS-PAGE loading buffer or Elution buffer (Pierce, 88848). For immunoblot analysis, cells were lysed with cell lysis buffer (CST, 9803) supplemented with a protease inhibitor ‘cocktail’. Protein concentrations in the extracts were measured by BCA assay (Pierce, 23235). Equal amounts of extracts were separated by SDS-PAGE, then transferred onto polyvinylidene fluoride membrane (Millipore, IPVH00010), blocked with 5% dry nonfat milk in Tris-buffered saline (pH 7.4) containing 0.1% Tween-20 and probed with the antibody for immunoblot analysis.

### Chromatin immunoprecipitation

Chromatin immunoprecipitation (ChIP) was performed by using a ChIP assay kit according to the manufacturer’s protocol (CST, 9003). Cells (2 × 10^7^) were cross-linked by formaldehyde. The ChIP-enriched DNA samples were quantified by real-time PCR, and the data are presented as the percentage of input. The primers used were as follows: forward 5′-ATTTTTAGCTCCGGGTCGGG-3′; reverse 5′-CGACGGCTCCTCCATCTTTG-3′.

### Dual-luciferase reporter assay

Dual-luciferase reporter assay was performed in 96-well plates by using a Dual-Luciferase/Renilla Reporter Assay System (Promega, Madison, WI) as described by the manufacturer. pRL-TK (Renilla luciferase) was used as an internal control. A total of 0.2 µg of DNA was used throughout.

### METABRIC dataset analysis

We evaluated overall survival (OS) defined as the intervals between the date of diagnosis for two genes HDAC5 and SOX9. We extracted normalised gene expression data for both genes from a public clinical database METABRICE (Molecular Taxonomy of Breast Cancer International Consortium). Expression data were analysed as continuous variables. We used Cox regression to quantify hazard ratio (HR) and the corresponding confidence interval (CI) in the models that included adjustment for age at diagnosis and TNM stage. This analysis was conducted by using SAS (version 9.4; SAS Institute, Inc., Cary, NC).^24^

### Statistical analysis

All data are representative of at least three independent experiments. All statistical analyses were performed by using Prism 8 (GraphPad). The data are presented as the mean ± SEM, and Student’s *t*-test was used for comparisons between two groups. *P*-value < 0.05 was considered statistically significant. Survival data were analysed by using Kaplan–Meier statistical method.

## Results

### SOX9 localised in the nucleus is required for tamoxifen resistance

**Fig. 1:**
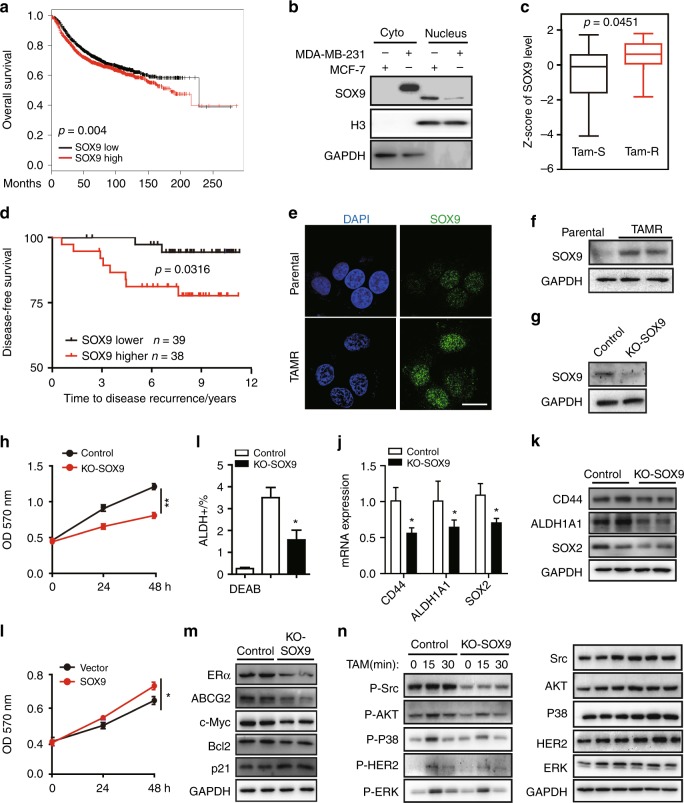
SOX9 localised in the nucleus is required for tamoxifen resistance. **a** Kaplan–Meier plot analysis of the correlation between the expression of SOX9 and survival rates in human breast cancer with public bioinformatics dataset (SOX9 low *n* = 1977, high *n* = 1974). **b** Western blot analysis of SOX9 protein of cytoplasmic and nuclear fractions from MDA-MB-231 and MCF-7 cells. **c** Comparison of SOX9 mRNA level in human breast cancer according to its sensitivities to tamoxifen by using GSE9195 cohort (ER^+^ breast cancer samples from women treated with adjuvant tamoxifen monotherapy, tamoxifen-sensitive *n* = 64 and tamoxifen-resistance *n* = 13). **d** Kaplan–Meier plot analysis of disease-free survival based on SOX9 mRNA levels by using GSE9195 cohort (ER^+^ breast cancer samples from women treated with adjuvant tamoxifen monotherapy, lower *n* = 39, higher *n* = 38). **e** Representative confocal images of immunofluorescence for SOX9 expression in MCF-7 and TAMR MCF-7 cells. Scale bar, 20 μm. **f** Western blot analysis for SOX9 protein in parental and TAMR MCF-7 cells. **g** Western blot for SOX9 protein in control and KO-SOX9 TAMR MCF-7 cells. **h** MTT assay of the growth rate in control or KO-SOX9 TAMR MCF-7 cells (lower). **i** Flow cytometric analysis of ALDH^+^ cells with control and SOX9-KO TAMR MCF-7 cells. **j** qRT-PCR of CD44, ALDH1A1 and SOX2 mRNA in TAMR MCF-7 cells with control or SOX9-KO. **k** Western blot analysis for CD44, ALDH1A1 and SOX2 proteins in TAMR MCF-7 cells with control or KO-SOX9. **l** MTT assay of growth rate after tamoxifen (1 μM) treatment in MCF-7 cells with SOX9 overexpression or vector control. **m** Western blot analysis for ERα C-MYC ABCG2 p21 and Bcl2 proteins in TAMR MCF-7 cells with control or KO-SOX9. **n** Western blot analysis of tamoxifen-resistance related signalling pathways in MCF-7 cells with control and SOX9-KO. Data are representative of means ± SEM of three independent experiments (unpaired *t-*test, *p < 0.05, **p < 0.01)

By analysis of the public available datasets, we observed that higher SOX9 expression is positively associated with poor survival rates in breast cancer patients^25^ (Fig. 1a). As SOX9 has distinct subcellular localisation in different types of breast cancer, so we first verified the localisation of SOX9 in ER-positive (MCF-7) and ER-negative (MDA-MB-231) breast cancer cells. The results showed that SOX9 was localised to the nucleus in ER^+^ MCF-7 breast cancer cells but to the cytoplasm in ER^–^ MDA-MB-231 breast cancer cells (Fig. 1b), suggesting that the localisation of SOX9 might be associated with different functions in ER^+^ or ER^–^ breast cancer. As tamoxifen treatment is an effective therapeutic way for ER^+^ breast cancer, we analysed the public datasets that focused on patients with tamoxifen treatment. By analysing the dataset GSE9195 cohort, we observed an increase in SOX9 transcription level in clinical samples of ER^+^ breast cancer that were resistant to tamoxifen compared with the sensitive ones (Fig. 1c). Higher SOX9 expression was significantly associated with poorer disease-free survival in tamoxifen-treatment patients (Fig. 1d). In order to investigate the expressions and localisation patterns of SOX9 in TAMR cells, we established two TAMR cell lines derived from MCF-7 and another ER^+^ cell line T47D by long-term tamoxifen treatment.^22,23^ We assessed the expressions of SOX9 in TAMR and parental MCF-7 or T47D. Immunofluorescence assay revealed increased nuclear localisation of SOX9 in TAMR cells (Fig. 1e). Elevated levels of SOX9 protein were found in TAMR cells compared with those in parental cells (Fig. 1f and Supplementary Fig. 1A).

To study the role of SOX9 in tamoxifen resistance, we further used CRISPR–cas9 assay to knock out SOX9 (KO-SOX9) in TAMR cells (Fig. 1g). Compared with control of TAMR MCF-7 and T47D cells, depletion of SOX9 restrained the growth of TAMR cells (Fig. 1h and Supplementary Fig. 1B). Previous studies have shown that breast CSC is crucial for tamoxifen resistance. SOX9 is an important transcription factor that confers CSC phenotype in breast cancer. We then investigated the proportions of CSCs in KO-SOX9 TAMR MCF-7 cells. The percentage of aldehyde dehydrogenase (ALDH)-positive cells, in which the activity of ALDH represents the tumour-initiating ability, decreased in KO-SOX9 TAMR MCF-7 cells (Fig. 1i). In addition, decreased expressions of CSC-related genes CD44, ALDH1A1 and SOX2 were also observed, confirming that SOX9 is required for the maintenance of breast CSCs in TAMR cells (Fig. 1j, k and Supplementary Fig. 1C). Consistently, MCF-7 with ectopically expressing SOX9 grew significantly faster than control cells when treated with tamoxifen (Fig. 1l). Similar results were observed in T47D TAMR cells with overexpressing SOX9 (Supplementary Fig. 1D). Next, we determined whether SOX9 could alter signalling pathways involved in tamoxifen resistance. Knocking out SOX9 in TAMR cells led to the decrease in some known contributing factors to endocrine resistance, such as ERα, drug transportation proteins of ABCG2, growth-related protein of Bcl2 and increase in apoptosis-related protein of p21 (Fig. 1m and Supplementary Fig. 1E). Furthermore, signalling pathways associated with tamoxifen resistance including HER2, Src and MAPK pathways, were also inhibited in SOX9-knockout MCF-7 cells (Fig. 1n). Taken together, these results indicate an important role of SOX9 in regulating the breast CSCs and tamoxifen resistance.

### HDAC5 is essential for the nuclear localisation of SOX9 in TAMR cells

**Fig. 2:**
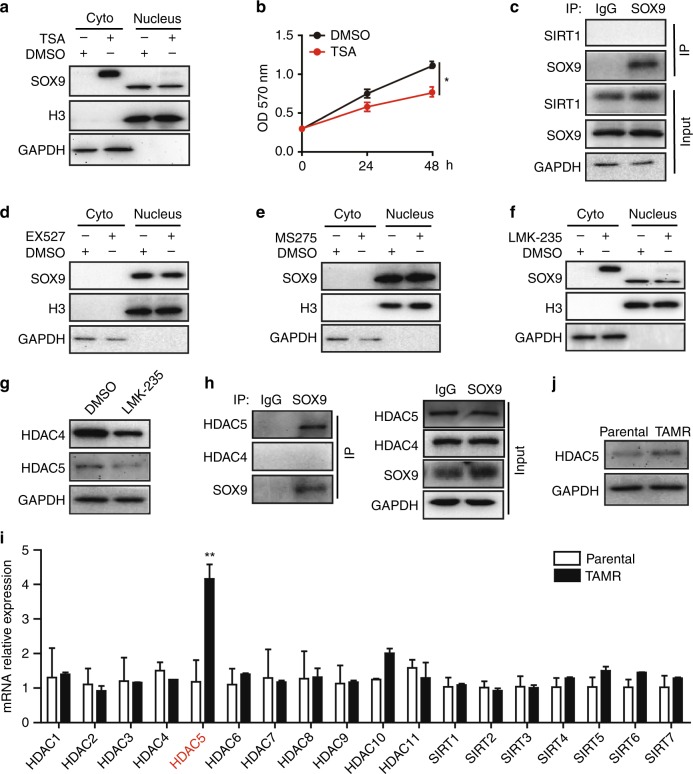
HDAC5 is essential for SOX9 nuclear localisation in TAMR cells. **a** Western blot analysis of SOX9 protein in cytoplasmic and nuclear fractions from DMSO or pan-HDAC inhibitor (TSA, 8 nM) treated with MCF-7 TAMR cells for 24 h. **b** MTT assay of growth rate of TAMR MCF-7 cells treated with DMSO or TSA (8 nM). **c** Immunoprecipitation of MCF-7 TAMR cells with anti-SOX9 antibody followed by immunoblotting with an antibody against SIRT1. **d** Western blot of SOX9 protein in cytoplasmic and nuclear fractions from MCF-7 TAMR cells treated with DMSO or SIRT1 inhibitor EX527 (30 μM). **e** Western blot of SOX9 protein in cytoplasmic and nuclear fractions from MCF-7 TAMR cells treated with DMSO or HDAC1/HDAC3 inhibitor MS275 (2 μM). **f** Western blot of SOX9 protein in cytoplasmic and nuclear fractions from MCF-7 TAMR cells treated with DMSO or HDAC4/HDAC5 inhibitor LMK-235 (12 nM). **g** Western blots of HDAC4 and HDAC5 proteins in TAMR cells treated with LMK-235 (12 nM). **h** Immunoprecipitation analysis in MCF-7 TAMR cells with anti-SOX9 antibody followed by immunoblotting with antibodies against HDAC4 and HDAC5. **i** HDACs 1~11 and SIRTs 1~7 mRNA expressions were analysed in parental and TAMR MCF-7 cells. **j** Western blot of HDAC5 protein in MCF-7 parental and TAMR cells. Data are representative of means ± SEM of three independent experiments (unpaired *t*-test, *p < 0.05)

It has been previously reported that SOX9 located in the cytoplasm abrogates the growth arrest response of HDAC inhibitors in basal-type breast cancer cells.^12^ To determine the effect of HDAC inhibitors on SOX9 localisation in TAMR cells, we used pan-HDAC inhibitor Trichostatin A (TSA) to treat TAMR cells. TSA treatment induced cytoplasm sequestration of SOX9 (Fig. 2a and Supplementary Fig. 1F) and inhibited TAMR cell growth (Fig. 2b and Supplementary Fig. 1G) both in MCF-7 and T47D TAMR cells. Moreover, the molecular weight of SOX9 in the cytoplasm was higher than that in the nucleus, suggesting that the post-transcriptional modification of SOX9 by HDACs is crucial for SOX9 nuclear localisation.

It has been identified that acetylation reduces SOX9 nuclear entry in chondrocytes. Sirtuin 1 (SIRT1) is crucial for SOX9 deacetylation in cartilage cells.^9^ We, therefore, detected whether SIRT1 was also involved in the deacetylation of SOX9 in TAMR MCF-7 cells. As shown, SOX9 could not interact with SIRT1 in TAMR MCF-7 cells (Fig. 2c). SIRT1 inhibitor EX527 treatment also did not change the SOX9 localisation in TAMR cells (Fig. 2d), indicating that SIRT1 was not involved in SOX9 deacetylation and nuclear translocation in TAMR MCF-7 cells.

We then treated TAMR MCF-7 cells with different HDAC inhibitors to identify which deacetylase was involved in SOX9 deacetylation. Treatment with MS275, an inhibitor of HDAC1 and HDAC3, failed to affect SOX9 nuclear localisation in TAMR cells (Fig. 2e). Interestingly, LMK-235, which is an HDAC4 and HDAC5 inhibitor, enhanced SOX9 cytoplasm localisation (Fig. 2f, g and Supplementary Fig. 1H). To further explore the key member of HDACs that mediates the deacetylation of SOX9, we used immunoprecipitation assay to identify the molecules that associate with SOX9. Given that LMK-235 inhibits the activations of HDAC4 and HDAC5, we then focused on HDAC4 and HDAC5. Immunoprecipitation analysis in MCF-7 TAMR cells showed that SOX9 interacted with HDAC5 but not HDAC4 (Fig. 2h). Interestingly, we compared the expression of 18 human deacetylation enzymes in MCF-7 parental and TAMR cells, in which only HDAC5 was significantly upregulated in TAMR cells compared with parental cells (Fig. 2i, j). Taken together, these results indicate that HDAC5 plays an important role in mediating SOX9 nuclear localisation in TAMR cells.

### HDAC5 is indispensable for deacetylation of SOX9 in TAMR cells

**Fig. 3:**
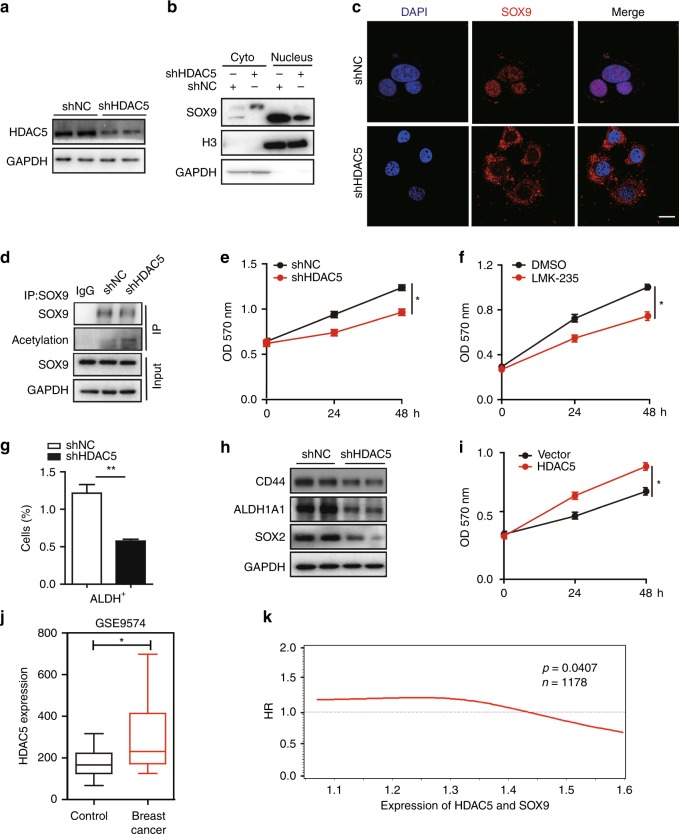
HDAC5 is indispensable for deacetylation of SOX9 in MCF-7 TAMR cells. **a** Western blot of HDAC5 protein from shNC and shHDAC5 TAMR MCF-7 cells. **b** Western blot of SOX9 protein in cytoplasmic and nuclear fractions from shNC and shHDAC5 MCF-7 cells. **c** Representative confocal images of immunofluorescence for SOX9 locations in shNC and shHDAC5 TAMR MCF-7 cells. **d** Immunoprecipitation in shNC and shHDAC5 MCF-7 cells with anti-SOX9 followed by immunoblotting with an antibody against acetylation proteins. **e** MTT assay of growth rates in shNC or shHDAC5 TAMR MCF-7 cells. **f** MTT assay of growth rates of TAMR MCF-7 cells treated with DMSO or HDAC4/HDAC5 inhibitor LMK-235 (12 nM). **g** Flow cytometry of ALDH^+^ cells following HDAC5 knocking down in TAMR MCF-7 cells. **h** Western blot analysis for CD44, ALDH1A1 and SOX2 proteins in TAMR MCF-7 cells with shNC and shHDAC5. **i** MTT assay of growth rates of MCF-7 transfected with HDAC5 or vector control and treated with tamoxifen (1 μM). **j** Bioinformatics analysis of HDAC5 expression in the public dataset (GEO: GSE9574) of healthy control and patients with ER^+^ breast cancer (control *n* = 14, breast cancer *n* = 14). **k** Cox regression to quantify hazard ratio (HR) and the corresponding confidence interval (CI) between overall survival (OS) and SOX9, HDAC5 expression in human breast cancer tissues (*N* = 1178, HR = 0.91, 95% CI: 0.82–1.0, *P* = 0.0409). Data are representative of means ± SEM of three independent experiments (unpaired *t-*test, *p < 0.05, **p < 0.01)

We have found that HDAC5 inhibitor could induce SOX9 cytoplasm localisation, prompting us to test whether HDAC5 is a major contributor for the acetylation and function of SOX9 in TAMR cells. Knocking down HDAC5 promoted cytoplasm localisation of SOX9 in MCF-7 and T47D TAMR cells (Fig. 3a, b and Supplementary Fig. 2A), which was also consistent with immunofluorescence staining results shown in Fig. 3c and Supplementary Fig. 2B. Significantly, knocking down HDAC5 increased the level of SOX9 acetylation by detecting the acetylation status of SOX9 with anti-acetylation antibody (Fig. 3d and Supplementary Fig. 2C). These results suggest that HDAC5 is dispensable for SOX9 deacetylation in TAMR cells.

We then further explored the function of HDAC5 in tamoxifen resistance. The growth of TAMR cells was significantly reduced after knocking down HDAC5 expression (Fig. 3e and Supplementary Fig. 2D). Similarly, LMK-235 treatment also reduced the growth of both MCF-7 and T47D TAMR cells (Fig. 3f and Supplementary Fig. 2E). Besides, knocking down HDAC5 also decreased the percentages of ALDH-positive cell population in TAMR cells (Fig. 3g). Consistent with the results from the SOX9-knockout cells, CSC-related molecules CD44, ALDH1A1 and SOX2 were reduced when knocking down HDAC5 in TAMR MCF-7 cells (Fig. 3h). Conversely, overexpression of HDAC5 in MCF-7 promoted the cell growth while treated with tamoxifen (Fig. 3i). Similar results were observed in T47D TAMR cells (Supplementary Fig. 2F). To examine the clinical relevance of HDAC5 in breast cancer, its expression values were analysed by using publicly available microarray data resources. Public dataset analysis (GEO: GSE9574) revealed that the expressions of HDAC5 in tumour tissues of ER^+^ breast cancer patients were higher than those in the epithelium adjacent to breast tumours (Fig. 3j). We further evaluated the associations between overall survival (OS) and two genes HDAC5 and SOX9 expression levels in endocrine-therapy-treated ER-positive cases from the METABRIC. The sample size is 1178 after excluding those participants who do not have TNM data. There are 503 deaths and the median survival is 7.53 years. We used combined genes of HDAC5 and SOX9 expression levels as an exposure in the calculation and found that the *P*-value was 0.0409 (95% CI: 0.82–0.99, *P* = 0.0409) (Fig. 3k). Thus, HDAC5 and SOX9 expression levels are crucial factors related to the overall survival in endocrine-therapy-treated ER^+^ breast cancer.

Collectively, these results suggest that HDAC5 deacetylates SOX9 to maintain its nuclear localisation, which drives tamoxifen resistance in breast cancer. HDAC5 and SOX9 are related to the poor survival rates in endocrine-therapy-treated ER^+^ breast cancer.

### HDAC5 directly interacts with SOX9

**Fig. 4:**
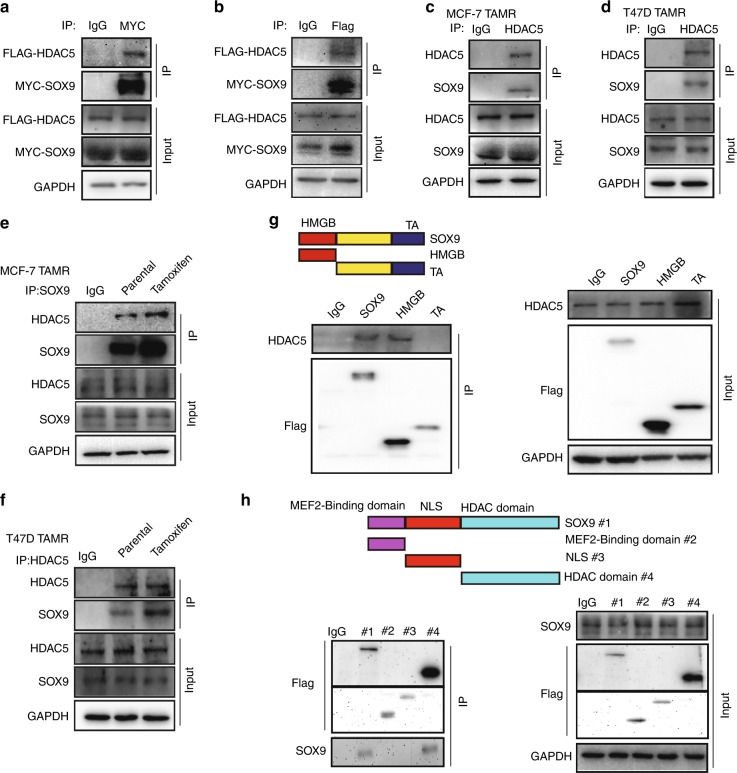
HDAC5 directly interacts with SOX9. **a** Co-immunoprecipitation in MCF-7 cells with anti-Flag HDAC5 followed by immunoblotting with an antibody against MYC–SOX9. **b** Co-immunoprecipitation assay in MCF-7 cells with anti-MYC SOX9 followed by immunoblotting with an antibody against Flag–HDAC5. **c** Immunoprecipitation in TAMR MCF-7 cells with anti-SOX9 followed by immunoblotting with an antibody against HDAC5. **d** Immunoprecipitation in TAMR T47D cells with anti-SOX9 followed by immunoblotting with an antibody against HDAC5. **e** Immunoprecipitation in parental and TAMR MCF-7 cells with anti-HDAC5 followed by immunoblotting with an antibody against SOX9. **f** Immunoprecipitation in parental and TAMR T47D cells with anti-HDAC5 followed by immunoblotting with an antibody against SOX9. **g** Co-immunoprecipitation assay in MCF-7 cells transfected with plasmid containing SOX9 HMGB domain or TA domain with anti-Flag-M2 beads followed by immunoblotting with an antibody against HA. Top: schematic diagram of full-length SOX9 and truncated mutants. **h** Co-immunoprecipitation assay in MCF-7 cells transfected with plasmid containing HDAC5 MEF2 binding domain or NLS domain, HDAC domain with anti-Flag-M2 beads followed by immunoblotting with an antibody against SOX9. Top: schematic diagram of full-length HDAC5 and truncated mutants. Scale bar, 20 μm. Data are representative of at least three independent experiments

Given that HDAC5 is critical for mediating the deacetylation of SOX9, we then tried to confirm that SOX9 directly interacts with HDAC5. The interaction between SOX9 and HDAC5 was observed in MCF-7 cells ectopically expressing SOX9 and HDAC5 (Fig. 4a, b). We next examined the interaction between endogenous SOX9 and HDAC5. SOX9 was shown to be associated with HDAC5 in MCF-7 and T47D TAMR cells (Fig. 4c, d). Thus, these results clearly indicated that there is an interaction between SOX9 and HDAC5 in TAMR cells. Moreover, stronger interaction between SOX9 and HDAC5 was found in TAMR cells than that in parental cells of MCF-7 and T47D (Fig. 4e, f). To identify which domain of SOX9 was required for its interaction with HDAC5, two truncated mutants of SOX9 were constructed: HMGB domain (1–181aa) mutant and c-terminal domain (TA domain) (182–507 aa) mutant.^26^ The results illustrated that HMGB domain of SOX9 is responsible for its interaction with HDAC5 (Fig. 4g). Then, the truncated mutants of HDAC5 were constructed: MEF2 binding domain (1–200 aa) mutant, NLS domain (200–498 aa) mutant and HDAC domain (498–1123 aa) mutant.^27^ The results indicated that HDAC domain of HDAC5 is critical for its interaction with SOX9 (Fig. 4h). Collectively, these data demonstrate that HDAC domain of HDAC5 binds to HMGB domain of SOX9 in TAMR cells.

### C-MYC directly promotes HDAC5 transcription

**Fig. 5:**
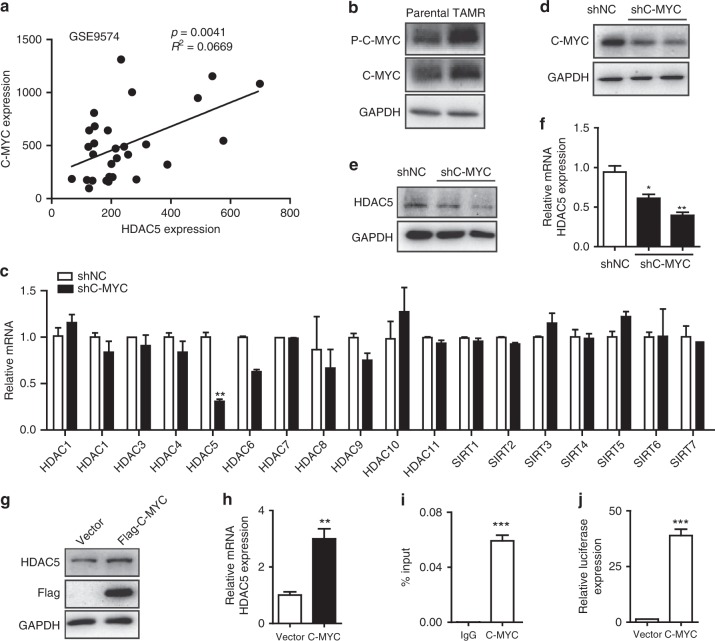
C-MYC promotes the expression of HDAC5. **a** Bioinformatics analysis of the public dataset (GEO: GSE9574) for the correlation between C-MYC and HDAC5 (*n* = 29, untreated ER^+^ breast cancers, *P* = 0.0041 and R squared = 0.2669). **b** Western blots of C-MYC and phosphorylated C-MYC (Ser62) in parental and TAMR MCF-7 cells. **c** qRT-PCR of HDAC1-11 and SIRT1-7 mRNAs in MCF-7 TAMR cells with shNC and shC-MYC. **d** Western blot of C-MYC in TAMR MCF-7 cells transfected with shC-MYC. **e** Western blot of HDAC5 in TAMR MCF-7 cells transfected with shC-MYC. **f** qRT-PCR of HDAC5 mRNA in TAMR MCF-7 cells with shNC and shC-MYC. **g** Western blot of Flag and HDAC5 in TAMR MCF-7 cells transfected Flag-C-MYC plasmid. **h** qRT-PCR of HDAC5 mRNA in TAMR MCF-7 cells transfected with Flag-C-MYC plasmid. **i** ChIP-qPCR analysis with the promoter of HDAC5 following the antibody against C-MYC in TAMR MCF-7 cells. **j** Dual-luciferase reporter assay for HDAC5 promoter in TAMR MCF-7 cells co-transfected C-MYC or control plasmid with Renilla Luciferase plasmids. Data are representative of means ± SEM of three independent experiments (unpaired *t-*test, *p < 0.05, **p < 0.01 and ***p < 0.001)

Although HDAC5 is highly expressed in TAMR cells and promotes tamoxifen resistance, the regulatory mechanism for HDAC5 upregulation in tamoxifen-resistant cells is not well understood. Online prediction tool PROMO was used to analyse the potential transcription factor-binding sites in HDAC5 promoter sequences. Among the 65 potential transcription factors, we speculated that C-MYC may be the key transcription factor for promoting HDAC5 transcription, as the expression of C-MYC is positively correlated with HDAC5 expression indicated by analysis with the public dataset GSE9574 in ER^+^ breast cancer (Fig. 5a). As it has been reported that C-MYC is crucial for tamoxifen resistance in breast cancer to overcome the growth reduction,^28^ we then measured C-MYC activation in TAMR MCF-7 cells. We confirmed that the protein levels of both C-MYC and phosphorylated C-MYC (Serine 62) were higher in MCF-7 TAMR cells compared with parental cells (Fig. 5b). When knocking down C-MYC in TAMR cells, HDAC5 expression was significantly reduced, while other class I and II HDACs and class III SIRTs were not affected (Fig. 5c–f). Conversely, ectopically expressing C-MYC in MCF-7 TAMR cells increased HDAC5 expression at both mRNA and protein levels (Fig. 5g, h).

Given that C-MYC expression is positively related with HDAC5 expression, we then aimed to explore whether C-MYC is a transcription factor for HDAC5 and directly regulates its expression. Sequence analysis showed that the promoter of HDAC5 contains the typical sequence of C-MYC binding sequence (CACGTG), which is located approximately −100 bp away from the upstream of the transcription start point. To further confirm the association between C-MYC and HDAC5, we used chromatin immunoprecipitation (CHIP) to detect whether C-MYC bound to the promoter of HDAC5. We designed primers for the region of −200/+20 bp upstream of the transcription start point of HDAC5. The results showed that C-MYC bound to the region of −200/+20 bp upstream of the transcription start point of HDAC5 in TAMR cells (Fig. 5i). The plasmid containing −200/+20 bp upstream of the transcription start point of HDAC5 was constructed, and consistent results were obtained from dual-reporter luciferase assay, which confirmed that C-MYC activates the expression of HDAC5 (Fig. 5j).

Collectively, these findings demonstrate that C-MYC is a transcription factor for HDAC5 and directly promotes its transcription in TAMR cells.

### C-MYC maintains SOX9 nuclear localisation in TAMR cells

**Fig. 6:**
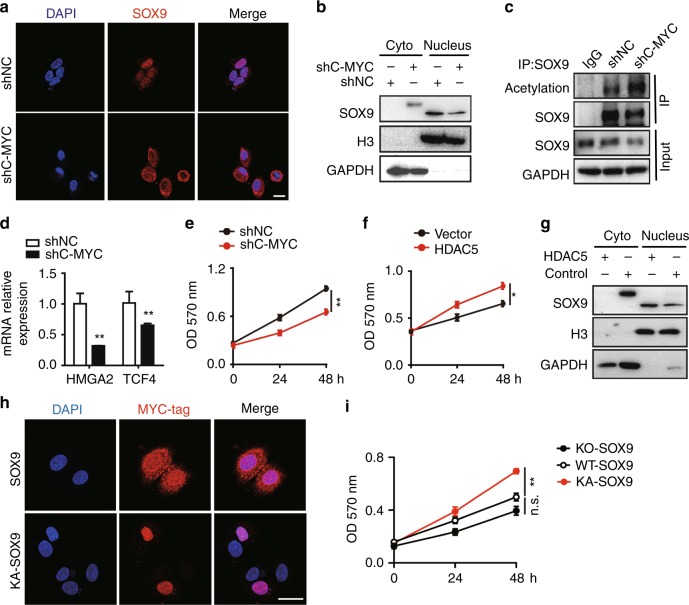
C-MYC maintains SOX9 nuclear localisation in TAMR MCF-7 cells. **a** Representative confocal images of immunofluorescence for SOX9 location of shC-MYC-MCF-7 TAMR cells. Scale bar, 20 μm. **b** Western blot of SOX9 protein in cytoplasmic and nuclear fractions from shNC or shC-MYC TAMR MCF-7 cells. **c** Immunoprecipitation in shNC or shC-MYC-MCF-7 TAMR cells with anti-SOX9 followed by immunoblotting with an antibody against the acetylation proteins. **d** qRT-PCR analysis of HMGA2 and TCF4 mRNAs in TAMR MCF-7 cells transfected with shNC and shC-MYC. **e** MTT assay of growth rates of TAMR MCF-7 cells with shNC or shC-MYC. **f** MTT assay of growth rates of shC-MYC TAMR MCF-7 cells overexpressing HDAC5 or control vector. **g** Western blot of SOX9 protein in cytoplasmic and nuclear fractions from shC-MYC TAMR MCF-7 cells transfected with HDAC5 or control vector. **h** Representative confocal images for MYC-SOX9 location in shC-MYC TAMR MCF-7 cells with overexpression of SOX9 or KA-SOX9. **i** Analysis of the growth rates of KO-SOX9 TAMR MCF-7 cells with overexpressing WT-SOX9 or KA-SOX9 vector by MTT assay. Scale bar, 20 μm. Data are representative of means ± SEM of three independent experiments (unpaired *t*-test, *p < 0.05, **p < 0.01)

Based on the fact that HDAC5 is indispensable for SOX9 nuclear localisation in TAMR cells, we speculated that C-MYC is required for the localisation and function of SOX9. Knocking down C-MYC inhibited SOX9 nuclear localisation that was shown by immunofluorescence staining in MCF-7 and T47D TAMR cells (Fig. 6a and Supplementary Fig. 3A). Nuclear and cytoplasm proteins were separated to confirm that SOX9 mostly localised in the cytoplasm in shC-MYC MCF-7 or T47D TAMR cells (Fig. 6b and Supplementary Fig. 3B). Then we used immunoprecipitation assay to detect the acetylation status of SOX9. Using anti-acetylation antibody for western blot revealed that knocking down C-MYC induced a higher acetylation level of SOX9 (Fig. 6c and Supplementary Fig. 3C). We next determined the effect of C-MYC on the expression of two SOX9 target genes, TCF4 and HMGA2. The results showed that there was significant reduction in the expression levels of these target genes when knocking down C-MYC in TAMR cells (Fig. 6d and Supplementary Fig. 3D). In addition, knocking down C-MYC reduced the growth of TAMR cells (Fig. 6e and Supplementary Fig. 3E). MTT assay showed that HDAC5 rescued the reducedgrowth rate in shC-MYC MCF-7 TAMR cells (Fig. 6f). Ectopically expressing HDAC5 in shC-MYC MCF-7 TAMR cells translocated SOX9 back into the nucleus (Fig. 6g and Supplementary Fig. 3F).

As reported, the deacetylation of 249 lysines of SOX9 is crucial for its nuclear localisation,^9^ we constructed full-length SOX9 with 249 lysine mutations replaced by glycine (KA-249). Transfecting KA-249 SOX9 into shC-MYC TAMR cells could restrict SOX9 to localising in the nucleus, while wild-type SOX9 was still localised in the cytoplasm (Fig. 6h). Transfecting KA-249 SOX9 rescued the SOX9-KO-induced growth suppression in MCF-7 TAMR cells but not the wild type of SOX9 (Fig. 6i).

Thus, these results indicate that C-MYC/HDAC5/SOX9 axis plays a critical role in maintaining tamoxifen resistance in breast cancer, which might be the potential target for reversing the tamoxifen resistance.

## Discussion

In this study, we provided the evidence that HDAC5 is indispensable for SOX9 deacetylation and nuclear translocation, and C-MYC is the transcription factor that directly promotes the expression of HDAC5. Therefore, C-MYC/HDAC5/SOX9 axis is essential for promoting tamoxifen resistance in breast cancer.

Protein acetylation, which was firstly found in histone lysine residues, mainly regulates gene transcription. Acetylation of non-histone proteins, such as p53, STAT3 and EZH2, also has been reported to play important roles in diverse physiological processes.^29–32^ Acetylation is a dynamic process and can be reversed by specific deacetylases. The opposite effects of histone acetyltransferases (HATs) and HDACs allow gene expression to be exquisitely regulated through chromatin remodelling and post-translational modifications.^33^ The modifications and localisation of SOX9 protein are important for its various functions in different cell types. The characteristics of distributions indicate that SOX9 has different functions between ER^+^ and basal-type breast cancers.^11,12^ SOX9 localised in the cytoplasm loses the function as a transcription factor for the expression of target genes. Here we report that SOX9 localisation is also affected by HDAC inhibitors in TAMR cells.

We treated TAMR cells with several selected inhibitors for HDACs and SIRTs. HDAC4 and HDAC5 inhibitor LMK-235 is able to render SOX9 to localise into the cytoplasm to decrease the proliferation of TAMR cells. We then identified that HDAC5 is critical for SOX9 nuclear localisation. In addition to SOX9, HDAC5 has also been previously reported to be able to deacetylate other non-histone proteins. In basal-type breast cancer, depletion of HDAC5 by shRNA not only hinders cell proliferation, manifesting as arresting G1 cell cycle, but also attenuates migration and colony formation of breast cancer cells through regulating LSD1 protein stability by decreasing LSD1 protein acetylation.^34^ Studies have also shown that deacetylase activity of HDAC5 is required for HIF-1α nuclear accumulation.^35^ In the present study, we found that HDAC5 is indispensable for SOX9 deacetylation. For clinical relevance, breast cancer patients with tamoxifen treatment expressing elevated SOX9 and HDAC5 have a worse overall survival. These results suggest that HDAC5 and SOX9 are involved in regulation of tumour progression after tamoxifen treatment. However, the correlation between SOX9 acetylation level and overall survival of tamoxifen-resistance breast cancer patients still needs more clinical evidence.

Although suitable endocrine therapy with tamoxifen or aromatase inhibitor can effectively increase the survival rate of breast cancers, studies have clarified many molecular mechanisms that cause endocrine-therapy resistance.^36^ There is an urgent requirement for an in-depth study of the relapse mechanisms for the endocrine therapy. Despite the promising clinical results produced by HDAC inhibitors in treatment of haematological malignancies such as T-cell lymphoma, no apparent clinical evidence indicates that HDAC inhibitors work effectively as a monotherapy against solid tumours including breast cancer.^19,37,38^ Here, we demonstrated that HDAC5 promotes the growth of TAMR cells and decreases the overall survival of breast cancer patients with tamoxifen treatment, which makes HDAC5 a potential therapeutic target for the reverse of tamoxifen resistance. The potential therapeutic value of combining tamoxifen with HDAC inhibitors is worth for clinical observation.

The complex interplays among transcription factors are essential for development and progression of breast cancer to a lethal disease.^6^ For example, the transcription factor FOXK2 represses the proliferation and invasion of breast cancer cells via reciprocal successive feedback by transcription factor HIF-1β.^24^ It has been shown that C-MYC is a crucial transcription factor for breast cancer progression and tamoxifen resistance.^39^ In this report we uncovered the connection between the transcription factors SOX9 and C-MYC. C-MYC increases the expression of HDAC5 that is dispensable for SOX9 deacetylation and tamoxifen resistance.

In summary, in the present study we identified HDAC5, whose transcription promoted by C-MYC, is essential for SOX9 deacetylation and nuclear localisation in tamoxifen-resistant breast cancer. Our novel findings also provide supportive evidence that an orchestrated interaction between HDAC5 and SOX9 is a critical mechanism to enhance transcriptional activities of tamoxifen-resistant related genes. Targeting C-MYC/HDAC5/SOX9 axis might be beneficial for the discovery of new strategies for ER^+^ breast cancer therapy.

## Supplementary information

supplementary file

## Data Availability

The datasets used and analysed during current study are available from the corresponding authors on reasonable request. The hyperlinks of publicly archived datasets involved in this study are as follows: https://www.ncbi.nlm.nih.gov/geo/query/acc.cgi?acc=GSE9195; https://www.ncbi.nlm.nih.gov/geo/query/acc.cgi?acc=GSE9574; http://molonc.bccrc.ca/aparicio-lab/research/metabric/

## References

[1] Ojo D, Wei F, Liu Y, Wang E, Zhang H, Lin X (2015). Factors promoting tamoxifen resistance in breast cancer via stimulating breast cancer stem cell expansion. Curr. Med. Chem..

[2] Jeselsohn R, Cornwell M, Pun M, Buchwalter G, Nguyen M, Bango C (2017). Embryonic transcription factor SOX9 drives breast cancer endocrine resistance. Proc. Natl Acad. Sci. USA.

[3] Abdelalim EM, Emara MM, Kolatkar PR (2014). The SOX transcription factors as key players in pluripotent stem cells. Stem. Cells Dev..

[4] Lefebvre V, Dvir-Ginzberg M (2017). SOX9 and the many facets of its regulation in the chondrocyte lineage. Connect. Tissue Res..

[5] Boiani M, Scholer HR (2005). Regulatory networks in embryo-derived pluripotent stem cells. Nat. Rev. Mol. Cell. Biol..

[6] Guo W, Keckesova Z, Donaher JL, Shibue T, Tischler V, Reinhardt F (2012). Slug and Sox9 cooperatively determine the mammary stem cell state. Cell.

[7] Larsimont JC, Youssef KK, Sanchez-Danes A, Sukumaran V, Defrance M, Delatte B (2015). Sox9 controls self-renewal of oncogene targeted cells and links tumor initiation and invasion. Cell Stem Cell.

[8] Castillo SD, Sanchez-Cespedes M (2012). The SOX family of genes in cancer development: biological relevance and opportunities for therapy. Expert Opin. Ther. Targets.

[9] Bar OzM, Kumar A, Elayyan J, Reich E, Binyamin M, Kandel L (2016). Acetylation reduces SOX9 nuclear entry and ACAN gene transactivation in human chondrocytes. Aging Cell.

[10] Liu C, Liu L, Chen X, Cheng J, Zhang H, Shen J (2016). Sox9 regulates self-renewal and tumorigenicity by promoting symmetrical cell division of cancer stem cells in hepatocellular carcinoma. Hepatology.

[11] Chakravarty G, Moroz K, Makridakis NM, Lloyd SA, Galvez SE, Canavello PR (2011). Prognostic significance of cytoplasmic SOX9 in invasive ductal carcinoma and metastatic breast cancer. Exp. Biol. Med..

[12] Chakravarty G, Rider B, Mondal D (2011). Cytoplasmic compartmentalization of SOX9 abrogates the growth arrest response of breast cancer cells that can be rescued by trichostatin A treatment. Cancer Biol. Ther..

[13] Amano Katsuhiko, Hata Kenji, Sugita Atsushi, Takigawa Yoko, Ono Koichiro, Wakabayashi Makoto, Kogo Mikihiko, Nishimura Riko, Yoneda Toshiyuki (2009). Sox9 Family Members Negatively Regulate Maturation and Calcification of Chondrocytes through Up-Regulation of Parathyroid Hormone–related Protein. Molecular Biology of the Cell.

[14] Dvir-Ginzberg, M., Gagarina, V., Lee, E. J. & Hall, D. J. Regulation of cartilage-specific gene expression in human chondrocytes by SirT1 and nicotinamide phosphoribosyltransferase. *J Biol Chem*. **283**, 36300–36310 (2008) 10.1074/jbc.M803196200 PMC2605985 18957417

[15] Lefebvre. V. & Dvir-Ginzberg. M. SOX9 and the many facets of its regulation in the chondrocyte lineage. *Connect Tissue Res*. **58**, 2–14 (2017). 10.1080/03008207.2016.1183667 PMC5287363 27128146

[16] Johnstone RW (2002). Histone-deacetylase inhibitors: novel drugs for the treatment of cancer. Nat. Rev. Drug Discov..

[17] Yang XJ, Seto E (2008). The Rpd3/Hda1 family of lysine deacetylases: from bacteria and yeast to mice and men. Nat. Rev. Mol. cell Biol..

[18] Urbich C, Rossig L, Kaluza D, Potente M, Boeckel JN, Knau A (2009). HDAC5 is a repressor of angiogenesis and determines the angiogenic gene expression pattern of endothelial cells. Blood.

[19] Duong V, Bret C, Altucci L, Mai A, Duraffourd C, Loubersac J (2008). Specific activity of class II histone deacetylases in human breast cancer cells. Mol. Cancer Res.: MCR.

[20] Huang WT, Tsai YH, Chen SH, Kuo CW, Kuo YL, Lee KT (2017). HDAC2 and HDAC5 up-regulations modulate survivin and mir-125a-5p expressions and promote hormone therapy resistance in estrogen receptor positive breast cancer cells. Front. Pharmacol..

[21] Huang Y, Vasilatos SN, Boric L, Shaw PG, Davidson NE (2012). Inhibitors of histone demethylation and histone deacetylation cooperate in regulating gene expression and inhibiting growth in human breast cancer cells. Breast Cancer Res. Treat..

[22] Raha P, Thomas S, Thurn KT, Park J, Munster PN (2015). Combined histone deacetylase inhibition and tamoxifen induces apoptosis in tamoxifen-resistant breast cancer models, by reversing Bcl-2 overexpression. Breast Cancer Res..

[23] Lu M, Ding K, Zhang G, Yin M, Yao G, Tian H (2015). MicroRNA-320a sensitizes tamoxifen-resistant breast cancer cells to tamoxifen by targeting ARPP-19 and ERRgamma. Sci. Rep..

[24] Shan L, Zhou X, Liu X, Wang Y, Su D, Hou Y (2016). FOXK2 Elicits Massive Transcription Repression and Suppresses the Hypoxic Response and Breast Cancer Carcinogenesis. Cancer cell.

[25] Gyorffy B, Lanczky A, Eklund AC, Denkert C, Budczies J, Li Q (2010). An online survival analysis tool to rapidly assess the effect of 22,277 genes on breast cancer prognosis using microarray data of 1,809 patients. Breast Cancer Res. Treat..

[26] Nikolova G, Vilain E (2006). Mechanisms of disease: Transcription factors in sex determination-relevance to human disorders of sex development. Nat. Clin. Pract. Endocrinol. Metab..

[27] Cao C, Vasilatos SN, Bhargava R, Fine JL, Oesterreich S, Davidson NE (2017). Functional interaction of histone deacetylase 5 (HDAC5) and lysine-specific demethylase 1 (LSD1) promotes breast cancer progression. Oncogene.

[28] Jin K, Park S, Teo WW, Korangath P, Cho SS, Yoshida T (2015). HOXB7 Is an ERalpha Cofactor in the Activation of HER2 and Multiple ER Target Genes Leading to Endocrine Resistance. Cancer Discov..

[29] Wan J, Zhan J, Li S, Ma J, Xu W, Liu C (2015). PCAF-primed EZH2 acetylation regulates its stability and promotes lung adenocarcinoma progression. Nucleic Acids Res..

[30] Yuan ZL, Guan YJ, Chatterjee D, Chin YE (2005). Stat3 dimerization regulated by reversible acetylation of a single lysine residue. Science.

[31] Kruse JP, Gu W (2008). SnapShot: p53 posttranslational modifications. Cell.

[32] Wan J, Xu W, Zhan J, Ma J, Li X, Xie Y (2016). PCAF-mediated acetylation of transcriptional factor HOXB9 suppresses lung adenocarcinoma progression by targeting oncogenic protein JMJD6. Nucleic Acids Res..

[33] Latham JA, Dent SY (2007). Cross-regulation of histone modifications. Nat. Struct. Mol. Biol..

[34] Vasilatos SN, Katz TA, Oesterreich S, Wan Y, Davidson NE, Huang Y (2013). Crosstalk between lysine-specific demethylase 1 (LSD1) and histone deacetylases mediates antineoplastic efficacy of HDAC inhibitors in human breast cancer cells. Carcinogenesis.

[35] Chen S, Yin C, Lao T, Liang D, He D, Wang C (2015). AMPK-HDAC5 pathway facilitates nuclear accumulation of HIF-1alpha and functional activation of HIF-1 by deacetylating Hsp70 in the cytosol. Cell Cycle.

[36] Dean M, Fojo T, Bates S (2005). Tumour stem cells and drug resistance. Nat. Rev. Cancer.

[37] Blumenschein GR, Kies MS, Papadimitrakopoulou VA, Lu C, Kumar AJ, Ricker JL (2008). Phase II trial of the histone deacetylase inhibitor vorinostat (Zolinza, suberoylanilide hydroxamic acid, SAHA) in patients with recurrent and/or metastatic head and neck cancer. Invest. New Drugs.

[38] Pili R, Liu G, Chintala S, Verheul H, Rehman S, Attwood K (2017). Combination of the histone deacetylase inhibitor vorinostat with bevacizumab in patients with clear-cell renal cell carcinoma: a multicentre, single-arm phase I/II clinical trial. Br. J. Cancer.

[39] Hynes NE, Stoelzle T (2009). Key signalling nodes in mammary gland development and cancer: Myc. Breast Cancer Res..

