# Supplementary material for: HDAC5-mediated deacetylation and nuclear localisation of SOX9 is critical for tamoxifen resistance in breast cancer
Source: Br J Cancer. 2019 Nov 6;121(12):1039–49. doi: 10.1038/s41416-019-0625-0 (PMC6964674; doi:10.1038/s41416-019-0625-0)
Supplement: Supplementary file 1 — supplementary file [file 41416_2019_625_MOESM1_ESM.docx]

**
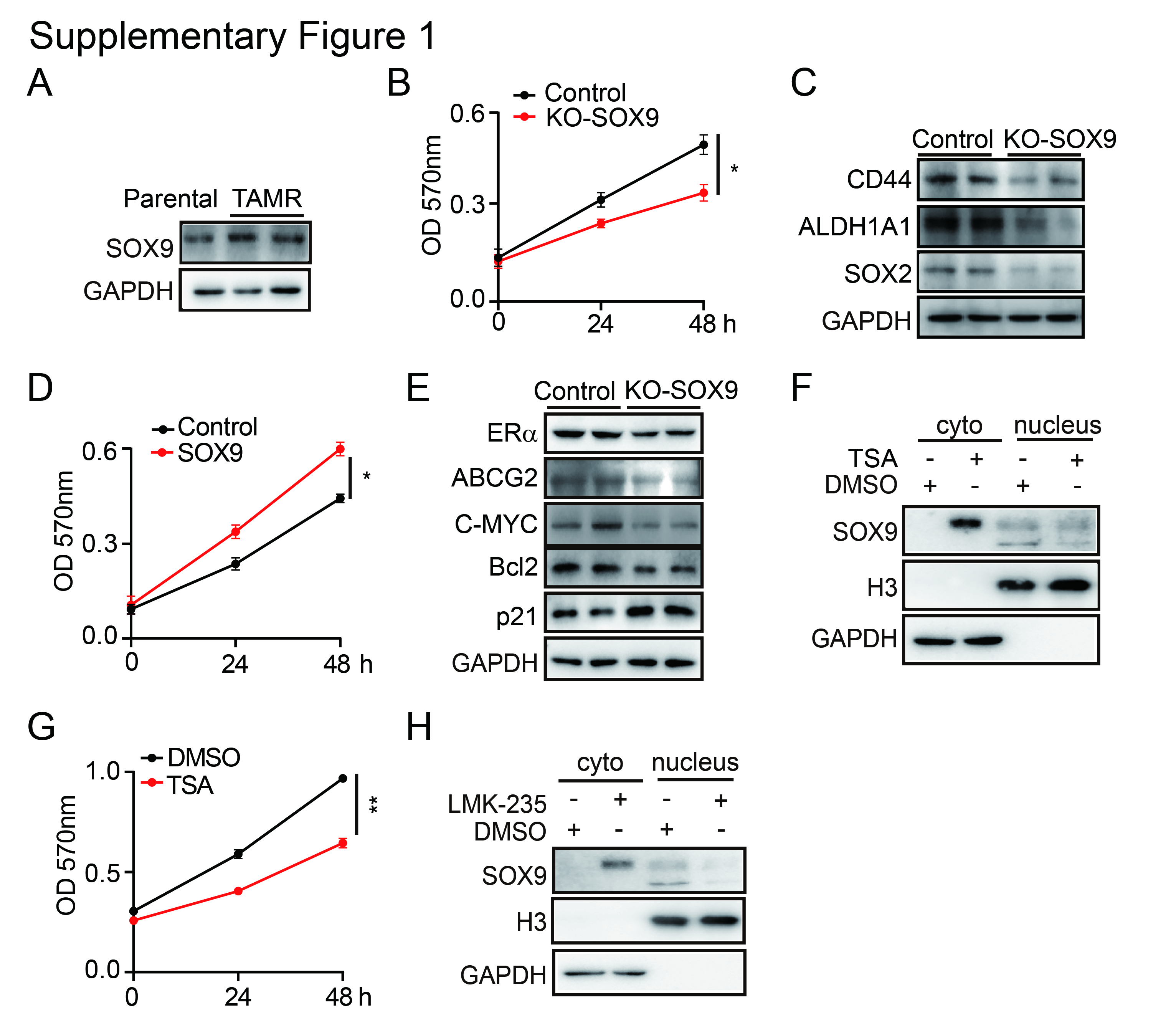
**

**Supplementary Figure 1. SOX9 localized in nucleus is required for tamoxifen resistance in T47D cell. (A)** Western blot analysis for SOX9 protein in parental and TAMR T47D cells. **(B)** MTT assay of growth rates in control or KO-SOX9 TAMR T47D cells. **(C)** Western blot analysis of CD44, ALDH1 and SOX2 proteins in TAMR T47D cells with control or SOX9-KO. **(D)** MTT assay of the growth rate of TAMR T47D cells with control or SOX9. **(E)** Western blot analysis for ERα, C-MYC, ABCG2, p21 and Bcl2 protein in TAMR T47D cells with control or KO-SOX9. **(F)** Western blot analysis of SOX9 protein in cytoplasmic and nuclear fractions from DMSO or pan-HDAC inhibitor (TSA, 8 nM) treated T47D TAMR cells for 24h. **(G)** MTT assay of the growth rate of TAMR T47D cells treated with DMSO or TSA (8 nM). **(H)** Western blot analysis of SOX9 protein in cytoplasmic and nuclear fractions from TAMR cells treated with DMSO or LMK-235 (12 nM). Data are representative of means ± SEM of three independent experiments (unpaired t test, *p<0.05，**p<0.01).

**
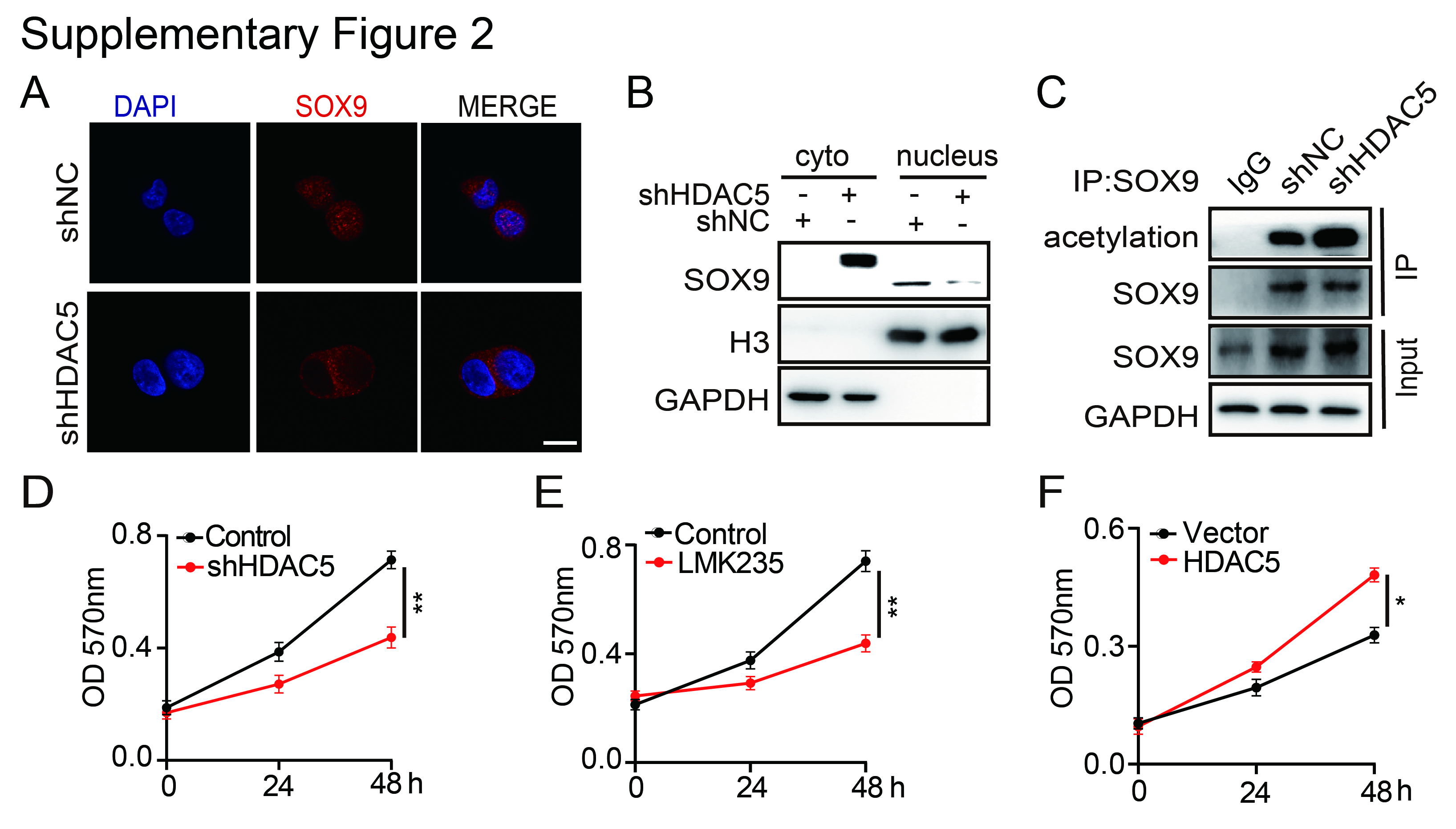
**

**Supplementary Figure 2. HDAC5 is indispensable for deacetylation of SOX9 in T47D TAMR cells.** **(A)** Representative confocal images of immunofluorescence for SOX9 locations in shNC and shHDAC5 TAMR T47D cells. **(B)** Western blot of SOX9 protein in cytoplasmic and nuclear fractions from shNC and shHDAC5 TAMR T47D cells. **(C)** Immunoprecipitation in shNC and shHDAC5 T47D cells with anti-SOX9 followed by immunoblotting with antibody against the acetylation proteins. **(D)** MTT assay of growth rates in shNC or shHDAC5 TAMR T47D cells. **(E)** MTT assay of growth rates of TAMR T47D cells with control or LMK235. **(F)** MTT assay of growth rates of TAMR T47D cells with control vector or HDAC5 overexpression. Data are representative of means ± SEM of three independent experiments (unpaired t test, *p<0.05，**p<0.01).

**
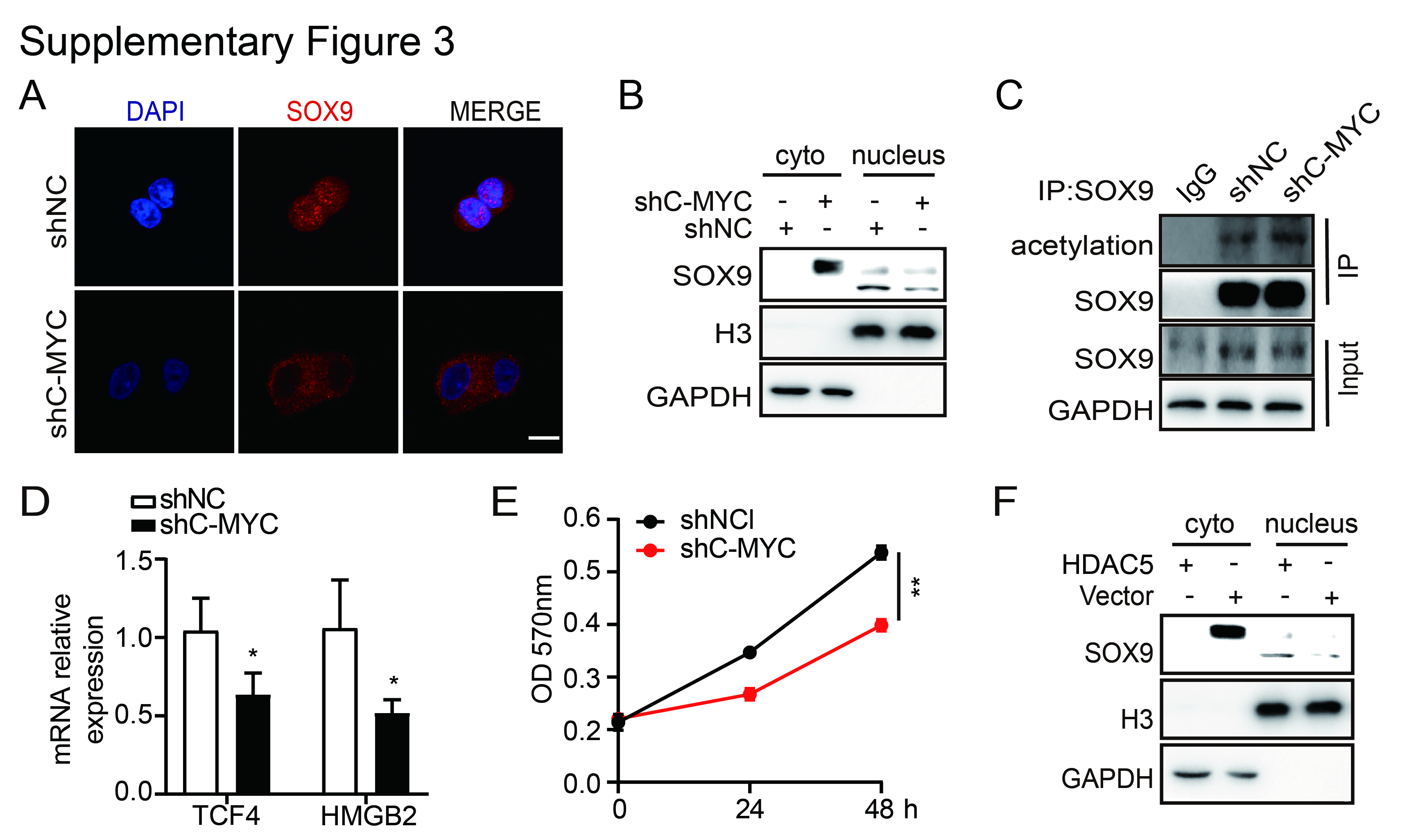
**

**Supplementary Figure 3. C-MYC maintains SOX9 nuclear localization in TAMR T47D cells. (A)** Representative confocal images of immunofluorescence for SOX9 location of shC-MYC-T47D TAMR cells. Scale bar, 20 μm. **(B)** Western blot of SOX9 protein in cytoplasmic and nuclear fractions from shNC or shC-MYC TAMR T47D cells. **(C)** Immunoprecipitation in shNC or shC-MYC-T47D TAMR cells with anti-SOX9 followed by immunoblotting with antibody against acetylation proteins. **(D)** qRT-PCR analysis of HMGA2 and TCF4 mRNAs in TAMR T47D cells transfected with shNC and shC-MYC. **(E)** MTT assay of growth rates of TAMR T47D cells with shNC or shC-MYC. **(F)** Western blot of SOX9 protein in cytoplasmic and nuclear fractions from shC-MYC TAMR T47D cells transfected with HDAC5 or control vector. Data are representative of means ± SEM of three independent experiments (unpaired t test, *p<0.05，**p<0.01).
